# EHMTI-0226. Prevalence of headache disorders in three different social settings

**DOI:** 10.1186/1129-2377-15-S1-D35

**Published:** 2014-09-18

**Authors:** ER Lebedeva, NR Kobzeva, TS Tsypushkina, PA Philimonova, DV Gilev, J Olesen

**Affiliations:** 1 Neurology, the Urals State Medical University, Yekaterinburg, Russia; 2 Neurology, Glostrup Hospital University of Copenhagen, Copenhagen, Denmark

## Introduction

The aim of our study was to estimate the prevalence of headache disorders in three different social settings using the newly published International Classification of headache Disorders(ICHD-3beta).

## Methods

The study population consisted of:1042 students (719 females, mean age 20.6, range 17-40), 1075 workers (146 females, mean age 40.4, range 21-67), 1007 blood donors (484 females, mean age 34.1, range 18-64). All patients were diagnosed according to ICHD-3beta using a semi-structured validated interview conducted by a neurologist or by trained senior medical students.

## Results

In the whole material, 1-year prevalence of headache was 67%, migraine 15% and tension type headache (TTH) 58%. In females the prevalence of migraine in students(35%) was significantly higher than in workers(16%) and blood donors(19%), p<0.0001.In males the prevalence of migraine in students(15%) was also significantly higher than in workers(4%) and donors(5%), p<0.0001. The prevalence of TTH in females was respectively 77%, 65% and 66%. In males the prevalence of TTH in students (79%) was significantly higher than in workers( 32% )and blood donors(59%), p<0.0001, the prevalence of TTH in donors (59%) was significantly higher than in workers(32%), p<0.0001. Only few (18%) had consulted because of headache. Particularly the very high prevalence of migraine and TTH in students is important and calls for further studies of risk factors and possibilities for preventive measures.

## Conclusions

We show for the first time convincingly that headache disorders have different prevalence according to social setting. They represent a huge health problem in Russia.

No conflict of interest.

